# Artificial arterial blood pressure artifact models and an evaluation of a robust blood pressure and heart rate estimator

**DOI:** 10.1186/1475-925X-8-13

**Published:** 2009-07-08

**Authors:** Qiao Li, Roger G Mark, Gari D Clifford

**Affiliations:** 1 Institute of Biomedical Engineering, School of Medicine, Shandong University, 44 Wenhua Xi Road, Jinan, Shandong, 250012, PR China; 2 Massachusetts Institute of Technology, 77 Massachusetts Avenue, Cambridge, MA, 02139, USA; 3 Harvard Medical School, 25 Shattuck Street, Boston, MA 02115, USA

## Abstract

**Background:**

Within the intensive care unit (ICU), arterial blood pressure (ABP) is typically recorded at different (and sometimes uneven) sampling frequencies, and from different sensors, and is often corrupted by different artifacts and noise which are often non-Gaussian, nonlinear and nonstationary. Extracting robust parameters from such signals, and providing confidences in the estimates is therefore difficult and requires an adaptive filtering approach which accounts for artifact types.

**Methods:**

Using a large ICU database, and over 6000 hours of simultaneously acquired electrocardiogram (ECG) and ABP waveforms sampled at 125 Hz from a 437 patient subset, we documented six general types of ABP artifact. We describe a new ABP signal quality index (SQI), based upon the combination of two previously reported signal quality measures weighted together. One index measures morphological normality, and the other degradation due to noise. After extracting a 6084-hour subset of clean data using our SQI, we evaluated a new robust tracking algorithm for estimating blood pressure and heart rate (HR) based upon a Kalman Filter (KF) with an update sequence modified by the KF innovation sequence and the value of the SQI. In order to do this, we have created six novel models of different categories of artifacts that we have identified in our ABP waveform data. These artifact models were then injected into clean ABP waveforms in a controlled manner. Clinical blood pressure (systolic, mean and diastolic) estimates were then made from the ABP waveforms for both clean and corrupted data. The mean absolute error for systolic, mean and diastolic blood pressure was then calculated for different levels of artifact pollution to provide estimates of expected errors given a single value of the SQI.

**Results:**

Our artifact models demonstrate that artifact types have differing effects on systolic, diastolic and mean ABP estimates. We show that, for most artifact types, diastolic ABP estimates are less noise-sensitive than mean ABP estimates, which in turn are more robust than systolic ABP estimates. We also show that our SQI can provide error bounds for both HR and ABP estimates.

**Conclusion:**

The KF/SQI-fusion method described in this article was shown to provide an accurate estimate of blood pressure and HR derived from the ABP waveform even in the presence of high levels of persistent noise and artifact, and during extreme bradycardia and tachycardia. Differences in error between artifact types, measurement sensors and the quality of the source signal can be factored into physiological estimation using an unbiased adaptive filter, signal innovation and signal quality measures.

## Background

Arterial blood pressure (ABP) is a basic hemodynamic parameter in intensive care unit (ICU) monitoring. ABP waveforms are frequently corrupted by artifacts, such as transducer flushing, catheter clotting, movement artifacts, and non-invasive cuff inflations [1]. These errors cause monitors to generate a high rate of false alarms. In fact, ICU false alarm rates can be as high as 86% [2,3]. Various strategies, such as median filtering [4], multi-parametric analysis [5-7], machine learning [8-11] and signal quality assessment techniques [12], are used to reduce false alarms.

Multiple average observations of blood pressure increase the accuracy of ABP estimates [13]. Therefore, a tracking procedure based upon some memory of previous values, that is not thrown off by individual errors should provide a more accurate method of estimating ABP. Furthermore, a system that can integrate an estimate of the quality of each individual observation into each ABP estimate can improve the overall ABP estimate further [12].

In this study we present extensions of our data fusion framework [14,15] which uses a robust Kalman filter (KF) and signal quality indices (SQI), for robust tracking of systolic blood pressure (SBP), mean blood pressure (MBP) and diastolic blood pressures (DBP) derived from ABP waveforms. After preliminary beat detection based on a localised slope in the low-pass filtered ABP signal [16], the signal quality of the ABP waveform is calculated by a combination of two previously developed SQI metrics, one using heuristic amplitude and gradient thresholds [17], and one using fuzzy representation and fuzzy reasoning [12]. Blood pressures are then estimated using a KF, adjusted to include the signal quality estimates.

## Methods

### Data sources

For the evaluation of our algorithm, we chose more than 6000 hours of high quality data (as judged by a stringent signal quality metric described in [12,17] and later in this article) comprising simultaneous ECG and ABP signals, from the Multi-Parameter Intelligent Monitoring for Intensive Care II (MIMIC II) database [18]. The MIMIC II database contains approximately 300,000 hours of bedside monitor waveform data from over 4,000 patients. Since no database of real ABP noise exists, we invented a series of ABP artifact simulation algorithms, and added these realistic artificial artifacts to clean ABP signals to create the evaluation data set.

### Artificial ABP artifact generation algorithms

After extensive searches through the MIMIC II database, we identified six generic phenomenological artifact types similar to those described in [1]. These are: 1) rapid saturation (over a period of 5 to 20 seconds) to some maximal ABP, 2) rapid saturation to some ABP minimum, 3) rapid saturation to the current mean ABP, 4) high amplitude square wave artifact, 5) high frequency noise and 6) highly transient impulse-like artifact. These artifacts are now described in more detail, with visual examples, and algorithms for generating realistic and variable artificial representations of them [Matlab source code for generating these artifacts, see Additional File 1]. Although the exact etiology of these artifact types is unknown, discussions with clinical staff who are familiar with these abnormal ABP waveform morphologies reveals that each artifact type is likely to be caused by damping (from a blocked arterial line), flushing of the arterial line (to reduce the damping), pinching of the arterial line, body movements or clinical activity or interventions. The resultant artifacts are discussed below in more detail with the mathematical definition of each artifact type.

#### 1. Saturation to ABP maximum artifact (a_smax_)

**Figure 1:**
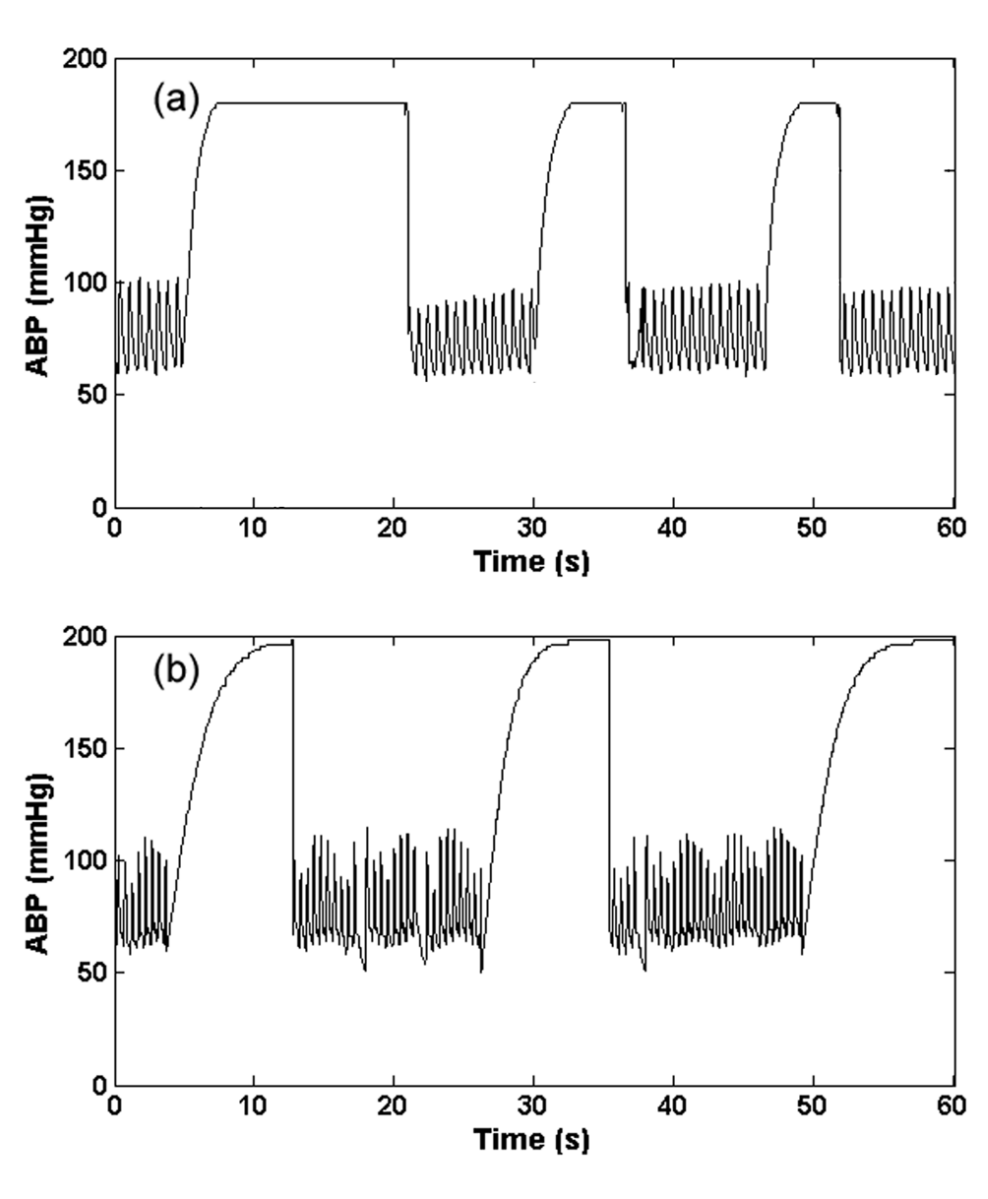
**Artifact 1, (*a*_smax_); Real (a) and simulated (b) ABP saturation to maximum pressure, defined by Eq. (1)**.

This type of artifact (as seen in Figure 1a) manifests as a rapid saturation from a normal ABP to a maximum value (*ABP*_max_), which is set to be equal to 200 mmHg ± 10 mmHg, with an exponential-like curve. We therefore use the hyperbolic tangent function (*tanh*) to simulate this behaviour as follows:

(1)

where *η*(0 <*η *≤ 1) is the rate of saturation, *A*_*dias *_is the diastolic ABP and *f*_*s *_is the sampling frequency of the ABP. A large value of *η *therefore leads to a rapid saturation to *ABP*_max_. Examples of the real and artificial *a*_smax _artifact are shown in Figures 1a and 1b respectively. This type of artifact is likely due to the flushing of the arterial line, to reduce damping caused by a blood clot or thrombosis of the arterial line, for example.

#### 2. Saturation to ABP minimum artifact (a_smin_)

**Figure 2:**
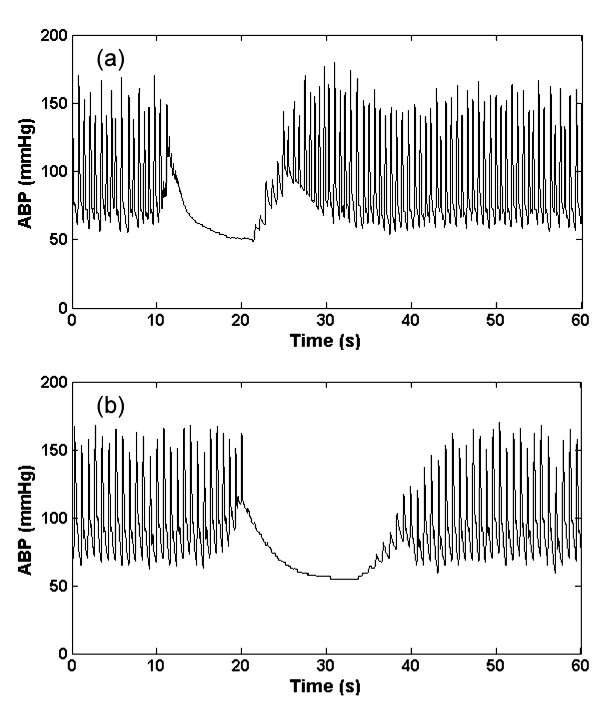
**Artifact 2, (*a*_smin_); Real (a) and Simulated (b) ABP saturation to lowest pressure in window defined by Eq.s (2) and (3)**.

This type of artifact (Figure 2) appears to be composed of four consecutive parts: 1) a rapid exponential diastolic saturation, 2) a rapid saturation from a normal ABP to a minimum value (*ABP*_min_) with an exponential-like decay, 3) an exponential increase from *ABP*_min _to some ABP value, and 4) a gradual transition back to the unaffected blood pressure. An artifact boundary is created with these four parts and then applied to the ABP. The upper boundary of the first part of the artifact is the maximum of ABP in the window, and the lower part is a right upside of a square function with the independent variable altered from 0 to 2.5. The upper boundary of the second part (*a*_smin_2_) is defined as:

(2)

And the lower part is obtained by decreasing *a*_smin_2 _to 90%. The upper boundary of the third and fourth part (*a*_smin_3_) is defined as:

(3)

where *N *is the length of the third and fourth parts. The lower third part is created by decreasing *a*_smin_3 _by 20% – 40% of its original value and the lower fourth part is the left downside of a square function. An example of the real and artificial *a*_smin _artifact is shown in Figures 2a and 2b respectively. This type of artifact may be due to a transient constriction in the arterial line such as pinching from arm movement.

#### 3. Reduced pulse pressure artifact (a_pp_)

**Figure 3:**
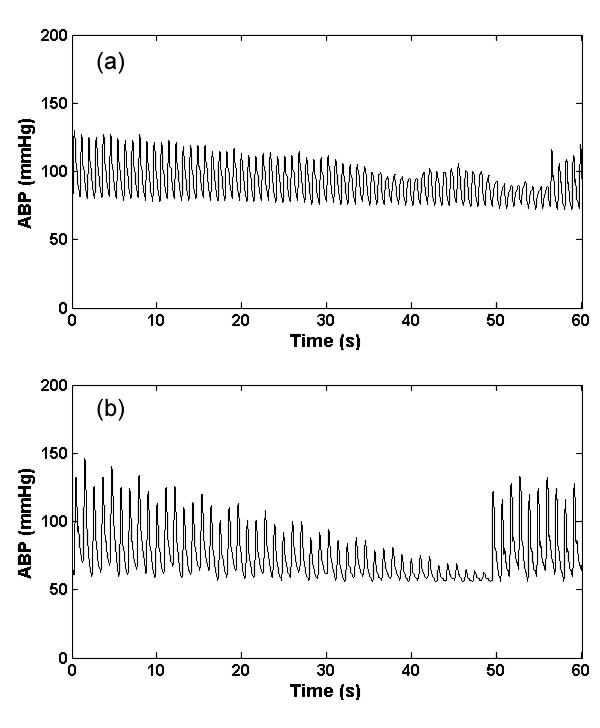
**Artifact 3, (*a*_pp_); Real (a) and Simulated (b) ABP reduced pulse pressure in window using a 5–10 Hz FIR filter plus Kaiser window**.

This type of artifact is similar to the systolic and diastolic ABP saturation artifact, gradually decreasing the pulse pressure. We simulated this artifact by decrementing the systolic ABP in a linear manner over a variable window length. A lowpass FIR filter with a passband cutoff of 5 Hz and a stopband cutoff of 10 Hz and a Kaiser window was also applied. An example of the real and artificial *a*_pp _artifact is shown in Figures 3a and 3b respectively. This type of artifact can be due to damping caused by thrombus in the arterial line.

#### 4. Square wave artifact (a_sw_)

**Figure 4:**
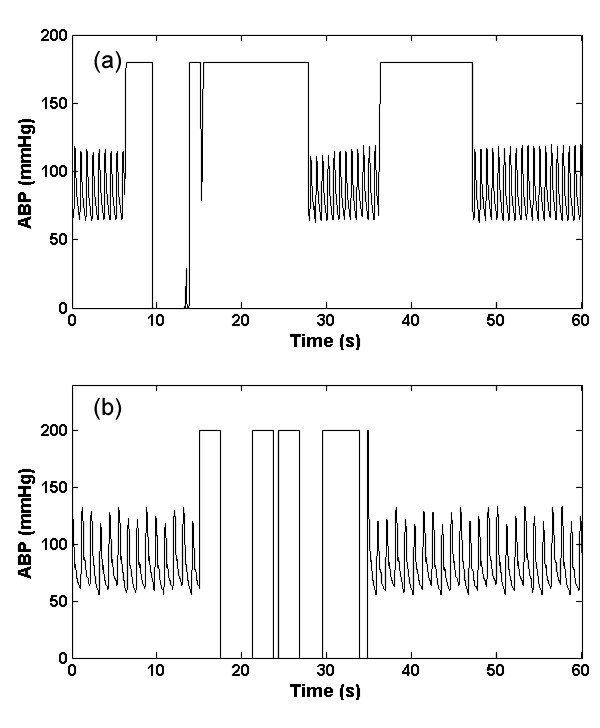
**Artifact 4, (*a*_sw_); Real (a) and Simulated (b) ABP square wave artifact**.

This type of artifact consists of a series of square waves with varying random duty cycles. Examples of the real and artificial *a*_sw _are shown in figure 4a and 4b. The length of the square wave is determined by generating a uniform random number distributed between 5 seconds and 20 seconds. This artifact simulates a fast flush test of the sensor [1]. A fast flush test helps determine the natural frequency and damping coefficient of the entire catheter monitoring system to ensure accuracy and consistency of the ABP measurement. Although a flush test is usually followed by a rapid oscillation that quickly dies away, this effect was not explicitly modeled here since it is brief and relatively low amplitude compared to the square wave. However, the addition of artifact type 6 (impulse artifact) to the end of each square pulse would allow modeling of this smaller effect.

#### 5. High frequency artifact (a_hf_)

**Figure 5:**
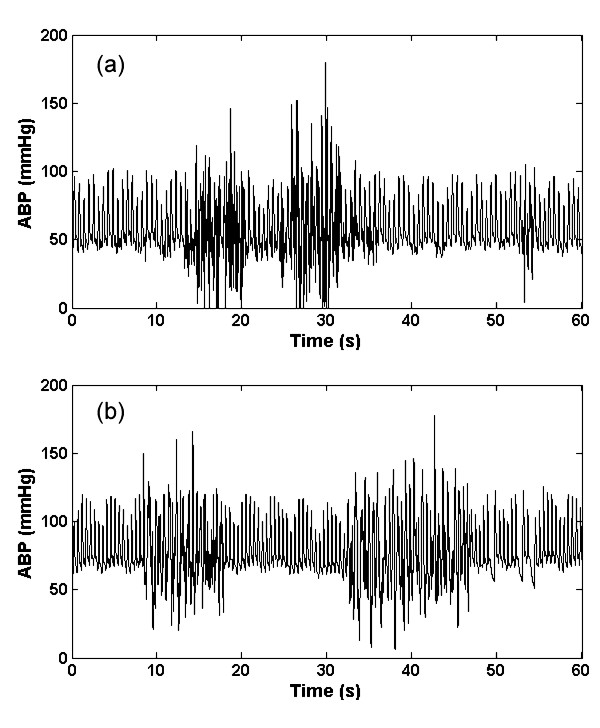
**Artifact 5, (*a*_hf_); Real (a) and Simulated (b) ABP high frequency artifact generated with band-limited (1.5 Hz to 18 Hz) 1/f^2 ^noise**.

This type of artifact is simulated by a brown noise generator (produced by Brownian motion), implemented through a 1/f^2 ^bandpass filter. The brown noise was further filtered using a Kaiser window FIR bandpass filter with a passband between 1.5 Hz to 18 Hz. The resultant signal was then added to the real ABP signals. Examples of the real and artificial *a*_hf _are shown in figures 5a and 5b respectively. High frequency noise can also be simulated by differentiating the signal. This band-pass filtering phenomenon may be related to movement artifact or disturbance of the transducer (such as dragging a cloth over the arterial line).

#### 6. Impulse artifact (a_imp_)

**Figure 6:**
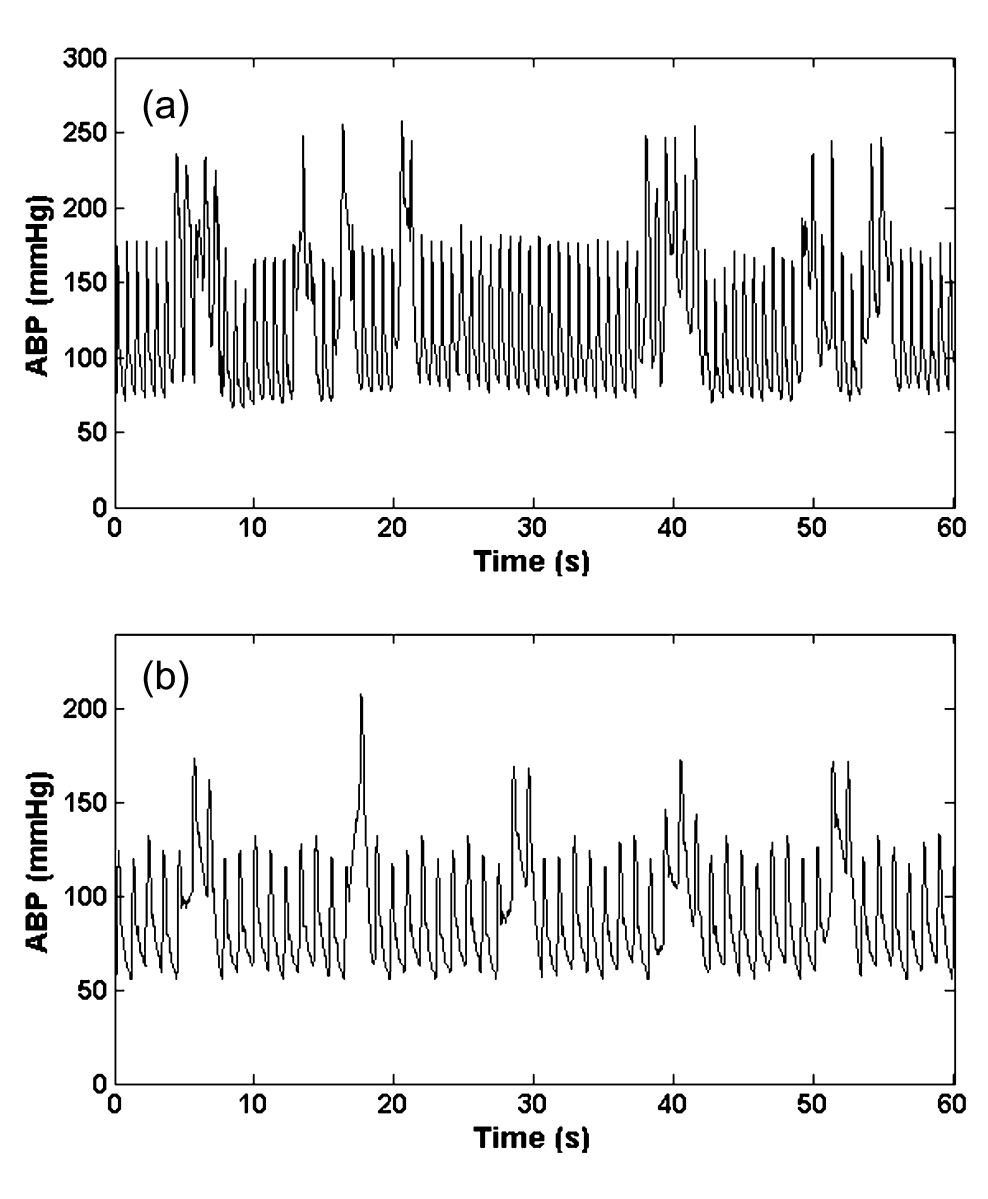
**Artifact 6, (*a*_imp_); Real (a) and Simulated (b) ABP impulse artefact using an adjustable sinc function (Eq. (4))**.

This type of artifact is simulated by the *sinc *function as shown in Eq. (4). The central lobe of the *sinc *function was used as *a*_imp _artifact and was added to the real ABP signals. The impulse artifact is given by

(4)

where *η*(0.05 <*η *< 0.35) is the approximate percentage of pulse that central lobe occupies, *n *defines the frequency at which the sinc function oscillates, and ⌊⌋ denotes selecting the central lobe of the *sinc *function. An example of the real and artificial *a*_imp _is shown in figures 6a and 6b respectively. This type of artifact could be due to motion, or a sharp mechanical artifact such as crimping of the tubing. It should be noted that these artifacts can also be applied to other biomedical signals such as the photoplethysmogram although the exact frequency response of each artifact and would require modification.

### Signal quality assessment

Two previously developed ABP signal quality assessment methods, *wSQI *[12] and *jSQI *[17] were combined to derive the signal quality index of ABP. The *wSQI *algorithm was designed to reduce the incidence of false ABP alarms by rejecting low-quality ABP segments. The algorithm consists of an open source ABP pulse onset detection routine, *wabp *[16], a waveform feature extraction routine, a waveform feature fuzzy representation, and a fuzzy reasoning procedure to produce the signal quality index. ABP waveform feature extraction was performed on a beat-by-beat basis. The waveform features used in this algorithm were systolic blood pressure (SBP), diastolic blood pressure (DBP), and mean blood pressure (MBP), maximum positive pressure slope, maximum negative pressure slope, maximum up-slope duration (the maximum duration over which the ABP signal can rise continuously), maximum duration above threshold (the maximum duration that the ABP signal stays above a pre-defined threshold), pulse-to-pulse interval (*T*), pulse pressure (the difference between the SBP and the DBP in a beat) and ECG-ABP delay time (the interval between the QRS onset in the ECG and the onset of the consequent ABP pulse).

The *wSQI *algorithm was previously trained on data from the MIMIC DB [19]. It was shown to give an accurate assessment of ABP signal quality in previous studies [12] with a sensitivity of 99.8% and a positive predictivity (positive predictive value) of 99.3% for detecting *true *blood pressure alarms and a sensitivity of 98.2% and a positive predictivity of 99.4% for detecting *false *alarms. A *wSQI *value is associated with each beat and possesses a continuous value between 0 and 1 (bad to good). Values for *wSQI *greater than 0.5 generally correspond to good signal quality (sufficient for heart rate analysis) [12].

The *jSQI *algorithm is a binary abnormality index (with 0 indicating a normal beat type) and uses the same beat detection algorithm as *wSQI*, after which features in each ABP pulse are identified (see table 1). Plausible heuristic constraints are set on the ABP amplitudes, slopes, and beat-to-beat variations in order to generate a signal abnormality index. The last column in table 1 lists these constraints. Note that for each beat, *P*_*d *_and *P*_*s *_are the local minimum and maximum around the pressure onset point. *P*_*m *_is the average pressure between adjacent onsets. *T *is the time difference between adjacent onsets. The first 5 criteria in Table 1 impose bounds on the physiologic ranges of each feature. For example, any beat with a diastolic pressure of less than 20 mmHg is flagged as abnormal.

**Table 1:** Features used in signal abnormality index jSQI. k is the beat index.

**Feature**	**Description**	**Abnormality CRITERIA**
*P*_*s*_	Systolic blood pressure (SBP)	*P*_*s *_> 300 mmHg

*P*_*d*_	Diastolic blood pressure (DBP)	*P*_*d *_< 20 mmHg

*P*_*m*_	Mean arterial pressure (MAP)	*P*_*m *_< 30 or *P*_*m *_> 200 mmHg

*f*	Instantaneous heart rate (60/T)	*f *< 20 or *f *> 200 BPM

*P*_*p*_	Pulse pressure (*P*_*s *_- *P*_*d*_)	*P*_*p *_< 20 mmHg

*w*	Noise term: mean of negative slopes	*w *< -40/100 mmHg/ms

*P*_*s*_[*k*] - *P*_*s*_[*k *- 1]	Absolute change in instantaneous SBP	|Δ*P*_*s*_| > 20 mmHg

*P*_*d*_[*k*] - *P*_*d*_[*k *- 1]	Absolute change in instantaneous DBP	|Δ*P*_*d*_| > 20 mmHg

*T*[*k*] - *T*[*k *- 1]	Absolute change in instantaneous HR	|Δ*T*| > 2/3 s

The sixth criterion in table 1 is the noise level, w, and is defined as the average of all negative slopes in each beat. With high frequency noise, there will be slopes with high negative gradients in the waveform. The final 3 criteria in table 1 compare ABP features between adjacent beats. Large sudden changes in beat-to-beat features are likely indications of abnormality. Each criterion is assigned a Boolean value for each beat, 0 for a normal range and 1 for an abnormal range (physiologically abnormal or noise/artifact). *jSQI *takes a binary value which is the logical *AND *of each 9 criteria. Compared to human annotation of signal quality, this algorithm has been shown to have a sensitivity of 1.00, and a positive predictivity of 0.91 [17].

The ABP signal quality index, *ψ*, is calculated by combining *wSQI *and *jSQI *as follows:

(5)

where 1 ≥ *η *≥ 0 is the positive coefficient chosen to be *η *= 0.7 and *jSQI *= 1 indicates an abnormal beat. That is, if *jSQI *indicates a good quality signal, *wSQI *can be believed. Otherwise, *wSQI *is trusted less, by a multiplicative coefficient, *η*, which effectively defines an arbitrary cut-off that defines the boundary between moderate and high quality data [14].

### Kalman filtering for tracking the ABP

#### 1. Kalman filtering algorithm

The KF is an optimal state estimation method for a stochastic signal [20,21] that estimates the state of a discrete-time controlled process, *x*, with measurement data *z*, where *x *and *z *are governed by the linear stochastic difference equations:

(6)

(7)

The random variables *w *and *v *are independent, white, and possess normal probability distributions, *p(w) ~ N(0, Q) *and *p(v) ~ N(0, R)*. The matrices ***A***, ***B***, ***H ***are the coefficient state transition matrices, ***Q ***being the state noise covariance, ***R ***the measurement noise covariance and *u *an optional control input to the state *x*.

The KF algorithm is given by the following equations:

(8)

(9)

(10)

(11)

(12)

where  and  are *a priori *and *a posteriori *state estimates before and after a given measurement *z*_*k*_;  and **P**_*k *_are the error covariances of *a priori *and *a posteriori *estimates,  is the measurement innovation (or residual); and ***K***_*k *_is the gain required to minimise the *a posteriori *error covariance, ***P***_*k*_.

We employed the KF to estimate the systolic, mean and diastolic blood pressure derived from the ABP at each pulse. However, in order to more heavily weight estimates derived from cleaner data, we propose the use of the SQI, *ψ*, to adjust the measurement noise covariance, ***R***, when ***K***_*k *_is updated. When the SQI is low, *z*_*k *_should be trusted less, so ***K***_*k *_should be small, and hence we force ***R ***to be large. This is achieved by modifying ***R ***as follows:

(13)

where ***R***_0 _is chosen to be equal to unity and is the unaltered value of ***R***. In other words, we do not assume the noise is stationary and instead the state noise covariance is adaptive based upon our signal quality measures.

It can be seen from Eq. (13) that this nonlinear transformation of ***R ***tends to unity as the SQI, *ψ*, tends to unity, and therefore doesn't affect the measurement noise covariance. When the SQI is high (near unity), the KF is forced to trust the current measurement, *z*_*k*_, and elevates the Kalman gain, ***K***_*k*_. At low values of *ψ*, ***R ***tends to infinity (but in practice is limited to a large value) and forces the KF to reduce ***K***_*k *_and hence trust the previous measurements more than the current measurement. Furthermore, an upper limit that defines the cusp between good and bad data, *ψ*_*t*_, is defined. When *ψ *<*ψ*_*t*_, the KF is not updated. The determination of the value of *ψ*_*t *_is performed in the same manner as for the ECG signal quality metrics described in [14]. This involves plotting the error with no SQI-control on the KF for each artifact type, and picking the value of the SQI that corresponds to an acceptable level of error for a particular application. In general, a value of *ψ*_*t *_= 0.5 provides an acceptable level of error (on average less than 20 mmHg). It should be noted that some types of artifact cause different levels of error and affect the SBP, MBP and DBP in different manners; see discussion.

#### 2. KF initialization and operation

Following Tarassenko and Townsend [22,23], we pick the simplest form of the KF, and set the state to be a scalar. (Scalar notation is therefore used from this point onwards.) Therefore we implemented three separate KFs, one for each of the SBP, MBP and DBP. We further assume that the blood pressure at each moment is approximately equal to the blood pressure at the next moment (*A≈1*). After neglecting the control input, Eq. (8) then reduces to . In order to initialise the KF, one must estimate *Q*, the state noise covariance matrix, and *R*, the measurement noise covariance, to calculate  and *K*_*k*_. *R *was similarly initialised to unity, noting that it is immediately modified by the SQI to reflect our trust in the data. *Q *was empirically adjusted to have an initial value of *Q *= 0.1. Values of *Q *< 0.1 lead to the KF trusting the state estimate too much and not adapting to the new initial observations. Values of *Q *> 0.1 lead to the KF trusting the new observations too much, and simply following the new values too closely. Setting *H *to unity then allows us to estimate the Kalman gain, *K*_*k*_, from Eq. (10) and hence the *a posteriori *error covariance estimate, *P*_*k*_, from Eq. (12). The filter can then be run online with only a few iterations (heart beats) for convergence. The Kalman residual is then given as  at each update (each detected beat).

### Methodology overview

The beat-onset detection method *wabp *[16] was applied to ABP waveform and ABP waveform features (SBP, MBP and DBP) were derived using the *wSQI *algorithm. The SQI for each beat was derived using a 10s window, centered on each beat. Second-by-second ABP features and SQI were acquired by calculating the median values of these beats within a moving 10s window, centered on each second. Then, the ABP values (SBP, MBP and DBP) together with the SQI, *ψ*, were inputted to the KF to obtain the optimal ABP estimation on a second-by-second basis. The final ABP values and SQI of each 10 second epoch were derived by calculating the median of the window's second-by-second output of the KF estimate of the ABP and SQI.

### Merging of multiple KF estimates; Dealing with missing and irregularly sampled data

In general it is possible to fuse any number of independent Kalman filtered observations, *X*, using the technique of Townsend and Tarassenko [22,23] such that

(14)

where *X*_*k *_is the *k*^th ^independent estimate and *σ*_*k *_is the innovation (equal to the residual, *r*_*k*_). In a recent paper [14] we proposed a modification to this approach where the SQI-scaled innovations are given by . In this way, when one channel (e.g. channel *k *= 1) is corrupted by artifact and the corresponding parameter estimate (*X*_1_) is miscalculated, the SQI (*SQI*_1_) will be low and the sudden change of *X*_1_, will make the residual error (*r*_1_) large. The weighted innovation () will therefore be large and the weighting for *X*_1_, (which would be  for two channels), will be small. The estimation of *X *will then rely more on *X*_2 _than *X*_1_.

In theory, each of the *X*_*k *_estimates can be recorded at different (or irregular/uneven) sampling frequencies. Adjustments to the innovation update sequence can be made to adjust for the differing sampling frequencies, and the inherent confidences in the different recording equipment. In general, the innovation-based weighting function can be modified so that

(15)

where 1 ≥ *λ*_*k *_≥ 0 is a 'trust' factor for the *k*^th ^channel of data. An example of the use of this parameter in blood pressure monitoring would be to fuse data from inflatable cuff measurements with direct arterial blood pressure. In this case, *λ*_*k *_for the ABP could be set to 1.0, and the sphygmomanometer-based cuff pressure can be set to be 0.8 (or some other relevant fractional value). In fact, the actual value of *λ*_*k *_can be calibrated for different BP measurement devices, which have been shown to produce significantly different errors [24-27]. For high pressure values, SBP is generally under-estimated by non-invasive cuff measurements (with respect to arterial measurements) [27] on average by 10 to 30 mmHg and DBP over-estimated on average by up to 10 mmHg [24]. At low blood pressures the opposite effect is seen [27]. It is also well-known that variation in protocol (such as arm position) can affect the accuracy of measurements [28]. Such knowledge can be incorporated into the confidence in the measure by adjusting *λ*_*k*_.

### Robust heart rate estimation

**Figure 7:**
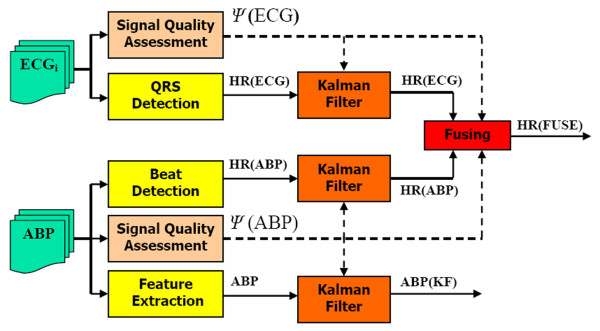
**General method for deriving heart rate and blood pressure from ICU signals**. Each channel of ECG and ABP is fed to beat detection and feature extraction algorithms. Signal quality, *ψ*, of each underlying signal is then performed. Derived parameters (HR and ABP) are fed to individual KFs, together with the signal quality of the channel of the channel from which the estimate is made. Finally, estimates of the same parameter are fused, using signal quality and the innovation from the KF to provide a robust estimate of the parameter.

Of course, a continuous blood pressure waveform also carries more information than just blood pressure. Together with ABP, heart rate estimation is extremely important as a first-order estimate of cardiovascular system performance. In a recent paper [14], we demonstrated how the combinatory approach detailed above can improve the heart rate estimate by fusing estimates from the ECG and ABP, with SQI-modified innovations. (The trust factors, *λ*_*k*_'s, were set to be equal to unity, to reflect the fact that, in the absence of signal quality information, we trust the ECG as much as the ABP signal to provide an estimate of heart rate.) Figure 7 illustrates the general logical flow of combining measures of HR and ABP from both the ECG and ABP waveform. Note that *ψ*(ECG) is the signal quality of the ECG as described in [14] and *ψ*(ABP) is the *ψ *described in this paper.

In other recent studies, we also used SQI-gated ABP signals to determine the validity of ICU arrhythmia alarms [6,7,29], demonstrating that the ABP waveform can make a significant difference to the accuracy of alarms when a signal quality index is used.

### Evaluation database

#### 1. Normal clean data

The ABP estimate algorithm was evaluated on the MIMIC II database, found at [18]. The following criteria were used to determine low-noise segments of the database: ECG signal quality [14] is good *and ψ ≥ 0.95 and *the length of the segment is greater than or equal to one hour in duration and at least one channel of ECG and ABP are simultaneously available for analysis. From a 2500 patient subset with 150,000 hours of available data, the resultant clean dataset included 437 cases, comprising 3762 one-hour or more (1.62 ± 0.69 hours) data segments, or 6084 hours total.

**Figure 8:**
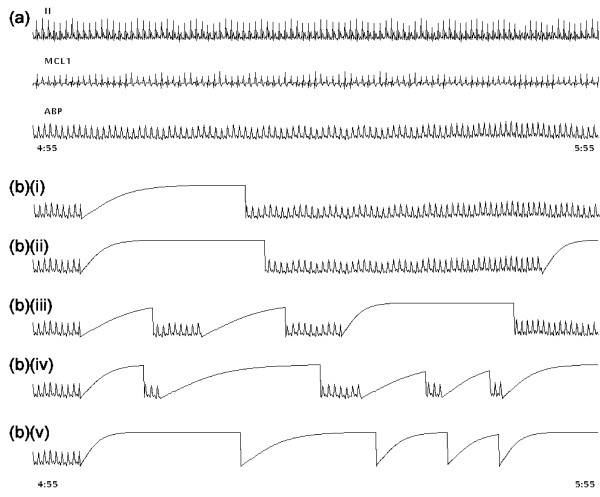
**Example of clean data and noisy ABP**. (a) clean data with 2 leads of ECG and 1 lead of ABP. (b) The same segment of ABP after adding artifact 1, (*a*_*smax*_), saturation to maximum ABP with (i) 20, (ii) 40, (iii) 60, (iv) 80 and (v) 100 percentage of noise duration. Note that the noisy segment begins 5s into the record at 5:00.

The six artificial ABP artifact models described above were then separately added to the clean dataset at different percentages of noise duration to generate the noisy evaluation dataset. In order to provide a periodic training period, the artifacts were only added to every other 5 minute epoch in the clean data. Therefore, in each hour, 6 noisy 5 minute periods are created each followed by 5 minutes of clean data. The percentage of each 5 minute noise segment containing artificial noise was set to 20%, 40%, 60%, 80% and then 100% for each experiment. That is, for each 5 minute segment that is designated to contain noise, artifacts are simulated and added at random (in a non-overlapping manner) to these respective portions of that 5 minute segment. Each noisy (5 minute data) segment was chose to follow a clean 5 minute segment. The rationale for this distribution was to allow the KF a period on which to train on the clean data. However, it turns out that much shorter segments of clean data could be chosen (see results section). Figure 8 illustrates the clean data and its appearance after adding the 'saturate to ABP maximum' artifact with differing percentages of artifact duration.

#### 2. Abnormal arrhythmia data

The ABP estimation algorithm was also evaluated on an abnormal data subset of the MIMIC II database, which includes episodes of annotated arrhythmias [7]. The presence of annotated arrhythmias allowed us to evaluate the performance of the algorithm for tracking heart rate changes during arrhythmic episodes which display sudden changes in HR and ABP. No gold standard, labelled blood pressure data is currently available, and so it was not possible to determine an independent objective measure for ABP during the arrhythmic episodes. However, in a related work [6,7] we created a database to aid the development of a false alarm suppression algorithm for the ICU. This database included a subset of over 5500 life-threatening alarms taken from the same MIMIC II database, for which we have simultaneous ECG and ABP data. A team of experts annotated each alarm (at least twice, with a separate pass and adjudication for disagreements), for the categories of asystole, extreme bradycardia, extreme tachycardia, ventricular tachycardia (VT) and ventricular flutter/fibrillation (VF). We do not consider the algorithm presented in this paper to be applicable to asystole, since asystoles shorter than 10 seconds would not be detected without parameter adjustments to the algorithm. Furthermore, ventricular arrhythmias are waveform morphology related, and therefore are not relevant to our HR-tracking algorithm presented here.

In the MIMIC II database there are over 45,000 hours of simultaneous ECG and ABP data with associated alarms of the above types. These data include 707 episodes of extreme bradycardia alarms (of which 506 are true and 201 false) and 1877 episodes of extreme tachycardia alarms (of which 1444 true and 433 false). Such false alarm rates (28.4% for bradycardia and 23.1% for tachycardia) are typical of alarms in the ICU, which can be as high as 85% [3], but for life threatening alarms are usually around 40% [7]. Epochs of 20 seconds around each extreme bradycardia and tachycardia alarm, (with the alarm occurring at 17 seconds in the epoch) were chosen providing a test set of 2584 events (1950 true episodes) and over 14 hours (861 minutes) of simultaneous ECG and ABP.

## Results

Following our previous work [14], we considered the Kalman filtered ABP of the clean dataset as the true ABP and a *gold standard *by which to evaluate the different ABP estimation methods. These were:

1. FE: Feature Extraction ABP estimate using *wSQI*.

2. SH: Sample-and-hold ABP estimate using ABP feature extraction routine of *wSQI *and clipping the reported ABP value when *ψ *<*ψ*_*t *_(SH). (This simulates the operation of monitoring equipment in the ICU.)

3. KF: ABP estimate using the SQI-based Kalman filter.

In order to evaluate the accuracy of an estimation method in the presence of noise, we chose the root mean squared error (rMSE) of the difference between each ABP estimation method and the true ABP. As expected, the rMSE is larger the lower the signal quality. Table 2] details the mean SQI, Ψ, ± one standard deviation, and the comparison of different ABP estimation methods on the whole evaluation dataset for each of the different types of artifacts detailed in section 2.1. Table 2 also provides these results for different percentages of artifact contamination. In general, for slow saturation artifacts, we can see that a value of *ψ *> 0.5 leads to a low ABP estimation error (less than 10 mmHg) using the SQI-based KF ABP estimation method described in this paper. However, the reduced pulse pressure artifact results in relatively large errors for the estimate of SBP when *ψ *≤ 0.9, indicating that we should trust the SBP less than the MBP or DBP, particularly in this circumstance. SBP estimates were corrupted mostly by saturations to ABP_min _and reduced pulse pressure. The MBP was mostly distorted by saturations to ABP_*max*_, square wave artifacts and saturations to ABP_min_.

**Table 2:** Ψ and ABP estimation error for different types of noise and percentage levels of noise.

Noise type	Noise percentages (%)	*Ψ*	Systolic ABP rMSE (mmHg)			Mean ABP rMSE (mmHg)			Diastolic ABP rMSE (mmHg)		
	
			FE	SH	KF	FE	SH	KF	FE	SH	KF
Saturate to ABP maximum (*a*_*s*max_)	20	0.86 ± 0.28	19.52 ± 18.15	3.92 ± 3.46	3.49 ± 3.21	29.55 ± 27.44	2.87 ± 2.46	2.38 ± 2.19	2.72 ± 2.43	2.49 ± 2.10	1.96 ± 1.85
	40	0.71 ± 0.31	26.64 ± 23.56	4.32 ± 3.66	3.72 ± 3.22	40.75 ± 35.47	3.19 ± 2.60	2.49 ± 2.21	3.25 ± 2.92	2.74 ± 2.24	2.03 ± 1.86
	60	0.53 ± 0.26	32.46 ± 27.11	4.97 ± 4.02	4.27 ± 3.67	49.79 ± 39.86	3.68 ± 2.86	2.73 ± 2.33	3.86 ± 3.44	3.08 ± 2.41	2.15 ± 1.92
	80	0.31 ± 0.20	41.20 ± 29.62	6.32 ± 4.72	5.52 ± 4.57	65.19 ± 39.41	4.93 ± 3.60	3.65 ± 3.03	4.57 ± 4.04	3.85 ± 2.76	2.68 ± 2.31
	100	0.18 ± 0.18	77.13 ± 37.37	6.78 ± 5.01	5.94 ± 4.90	91.15 ± 41.62	5.07 ± 3.56	3.96 ± 3.30	24.33 ± 19.98	4.14 ± 2.90	2.89 ± 2.48

Saturate to ABP minimum (*a*_*s*min_)	20	0.90 ± 0.32	38.13 ± 34.84	7.33 ± 6.90	6.00 ± 5.59	22.88 ± 21.36	2.09 ± 1.83	1.73 ± 1.54	16.22 ± 15.27	1.33 ± 1.12	1.18 ± 1.05
	40	0.82 ± 0.38	50.03 ± 42.85	9.29 ± 8.58	7.66 ± 6.91	30.20 ± 26.91	2.46 ± 2.09	2.07 ± 1.76	21.49 ± 19.41	1.55 ± 1.25	1.41 ± 1.19
	60	0.73 ± 0.40	59.62 ± 47.45	11.07 ± 9.98	9.15 ± 7.92	36.11 ± 30.57	2.91 ± 2.42	2.46 ± 2.02	25.74 ± 22.33	1.81 ± 1.42	1.65 ± 1.33
	80	0.64 ± 0.39	67.88 ± 49.51	12.94 ± 11.08	11.10 ± 8.65	41.19 ± 32.93	3.29 ± 2.65	2.84 ± 2.22	29.43 ± 24.13	2.11 ± 1.57	1.95 ± 1.43
	100	0.54 ± 0.36	75.20 ± 49.03	17.61 ± 12.44	17.80 ± 10.66	45.67 ± 34.24	3.97 ± 2.98	3.88 ± 2.70	32.70 ± 25.06	3.46 ± 2.02	3.37 ± 1.93

Reduced pulse pressure (*a*_PP_)	20	0.92 ± 0.19	16.71 ± 14.75	12.04 ± 10.54	10.69 ± 9.33	5.80 ± 5.01	4.33 ± 3.67	3.69 ± 3.21	2.27 ± 1.90	2.40 ± 2.00	1.96 ± 1.83
	40	0.87 ± 0.22	21.84 ± 17.81	15.62 ± 12.67	13.81 ± 11.06	7.38 ± 6.01	5.32 ± 4.26	4.47 ± 3.65	2.42 ± 1.97	2.58 ± 2.08	2.05 ± 1.86
	60	0.81 ± 0.23	25.95 ± 19.23	18.61 ± 13.74	16.44 ± 11.83	8.66 ± 6.58	6.16 ± 4.61	5.14 ± 3.92	2.57 ± 2.03	2.75 ± 2.13	2.14 ± 1.89
	80	0.76 ± 0.23	29.54 ± 19.36	21.71 ± 14.03	19.38 ± 11.82	9.77 ± 6.86	6.96 ± 4.81	5.83 ± 4.11	2.71 ± 2.07	2.90 ± 2.17	2.24 ± 1.91
	100	0.72 ± 0.22	33.21 ± 18.25	26.10 ± 13.80	24.41 ± 12.09	10.80 ± 6.93	8.03 ± 5.04	7.06 ± 4.41	2.78 ± 2.11	3.01 ± 2.20	2.34 ± 1.95

Square wave artifact (*a*_sw_)	20	0.86 ± 0.28	19.38 ± 18.26	3.69 ± 3.21	3.23 ± 2.95	9.03 ± 8.50	2.77 ± 2.35	2.24 ± 2.06	3.91 ± 3.62	2.44 ± 2.06	1.91 ± 1.79
	40	0.71 ± 0.31	26.54 ± 24.07	4.04 ± 3.37	3.42 ± 3.02	12.09 ± 11.10	3.07 ± 2.47	2.34 ± 2.08	4.93 ± 4.58	2.71 ± 2.21	1.99 ± 1.80
	60	0.53 ± 0.26	32.43 ± 28.15	4.57 ± 3.63	3.81 ± 3.24	14.63 ± 13.05	3.49 ± 2.65	2.54 ± 2.19	5.82 ± 5.37	3.05 ± 2.38	2.13 ± 1.86
	80	0.31 ± 0.19	43.10 ± 32.44	6.38 ± 4.73	5.63 ± 4.61	18.49 ± 15.14	4.80 ± 3.39	3.76 ± 3.12	6.73 ± 6.08	3.93 ± 2.81	2.79 ± 2.38
	100	0.18 ± 0.17	73.95 ± 33.22	6.75 ± 4.97	5.94 ± 4.90	33.09 ± 21.47	5.04 ± 3.53	3.96 ± 3.31	20.10 ± 13.67	4.13 ± 2.90	2.90 ± 2.48

High frequency artifact (*a*_hf_)	20	0.90 ± 0.17	3.84 ± 3.35	4.03 ± 3.52	3.58 ± 3.15	2.54 ± 2.18	2.78 ± 2.38	2.68 ± 2.28	6.85 ± 6.01	5.79 ± 5.05	4.93 ± 4.14
	40	0.82 ± 0.17	4.38 ± 3.69	4.64 ± 3.91	4.09 ± 3.36	2.74 ± 2.29	3.06 ± 2.54	3.17 ± 2.39	9.32 ± 7.56	8.12 ± 6.57	6.96 ± 5.01
	60	0.73 ± 0.14	4.91 ± 3.97	5.26 ± 4.24	4.74 ± 4.35	2.95 ± 2.39	3.36 ± 2.70	3.76 ± 2.47	11.55 ± 8.31	10.25 ± 7.34	9.44 ± 5.46
	80	0.66 ± 0.12	5.76 ± 4.36	6.27 ± 4.73	6.10 ± 4.35	3.35 ± 2.51	3.92 ± 2.94	4.96 ± 2.76	15.20 ± 8.42	13.62 ± 7.58	14.59 ± 6.72
	100	0.62 ± 0.13	7.67 ± 5.16	7.88 ± 5.37	7.59 ± 5.18	4.19 ± 2.79	4.69 ± 3.21	6.23 ± 3.21	19.35 ± 8.85	16.90 ± 8.39	18.82 ± 8.59

Impulse artifact (*a*_imp_)	20	0.93 ± 0.16	5.58 ± 4.91	3.88 ± 3.42	3.33 ± 3.06	5.51 ± 4.87	3.47 ± 3.01	2.67 ± 2.44	4.28 ± 3.76	3.42 ± 2.98	2.46 ± 2.28
	40	0.85 ± 0.19	7.24 ± 6.05	4.43 ± 3.79	3.68 ± 3.28	7.45 ± 6.21	4.33 ± 3.62	3.17 ± 2.80	5.70 ± 4.81	4.40 ± 3.73	3.02 ± 2.68
	60	0.77 ± 0.18	8.43 ± 6.42	5.02 ± 4.12	4.03 ± 3.49	8.87 ± 6.54	5.23 ± 4.11	3.70 ± 3.15	6.81 ± 5.29	5.25 ± 4.22	3.60 ± 3.06
	80	0.69 ± 0.15	9.52 ± 6.61	5.59 ± 4.39	4.49 ± 3.73	10.18 ± 6.69	6.05 ± 4.44	4.36 ± 3.43	7.72 ± 5.55	6.05 ± 4.60	4.24 ± 3.35
	100	0.66 ± 0.18	13.29 ± 7.58	7.59 ± 5.59	6.41 ± 4.97	14.22 ± 7.04	8.58 ± 5.68	6.95 ± 4.89	10.96 ± 6.62	8.89 ± 6.02	7.13 ± 4.99

**Figure 9:**
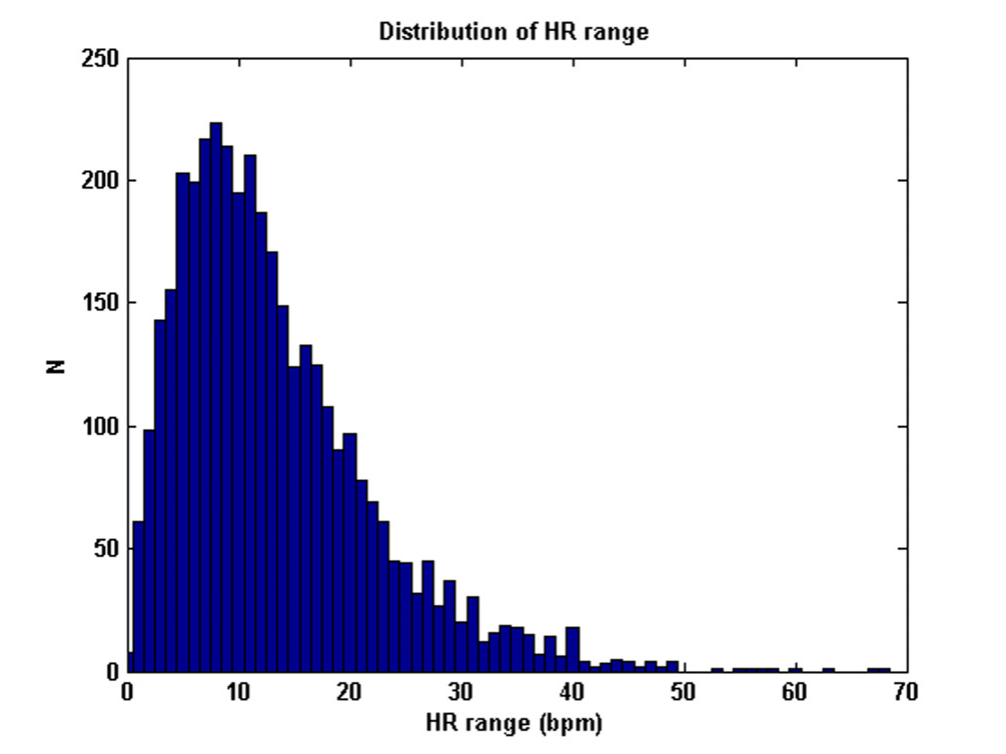
**The distribution of true HR range (HR_max_-HR_min_) of the clean dataset comprising 3762 data segments of 1 hour or longer continuous waveform data (1.62 ± 0.69 h)**.

**Figure 10:**
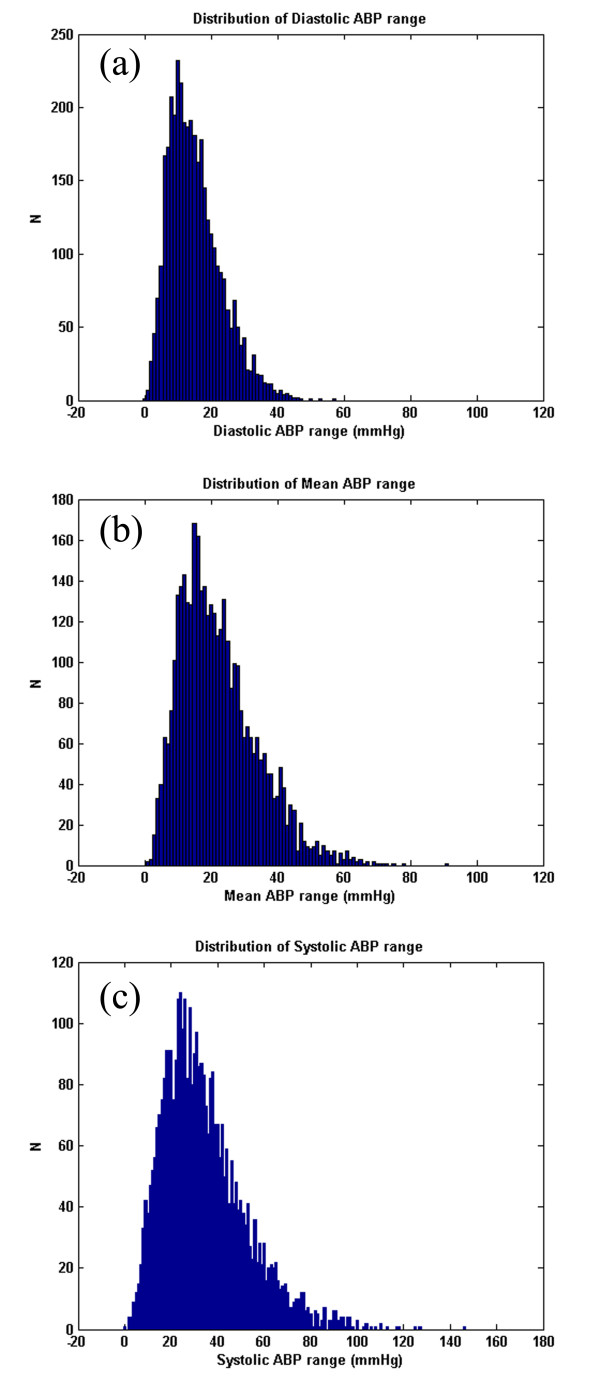
**The distribution of true BP range (ABP_max_-ABP_min_) of the clean dataset for the DBP(a), MBP(b) and SBP(c) for each segment of data comprising 3762 data segments of 1 hour or longer continuous waveform data (1.62 ± 0.69 h)**.

**Figure 11:**
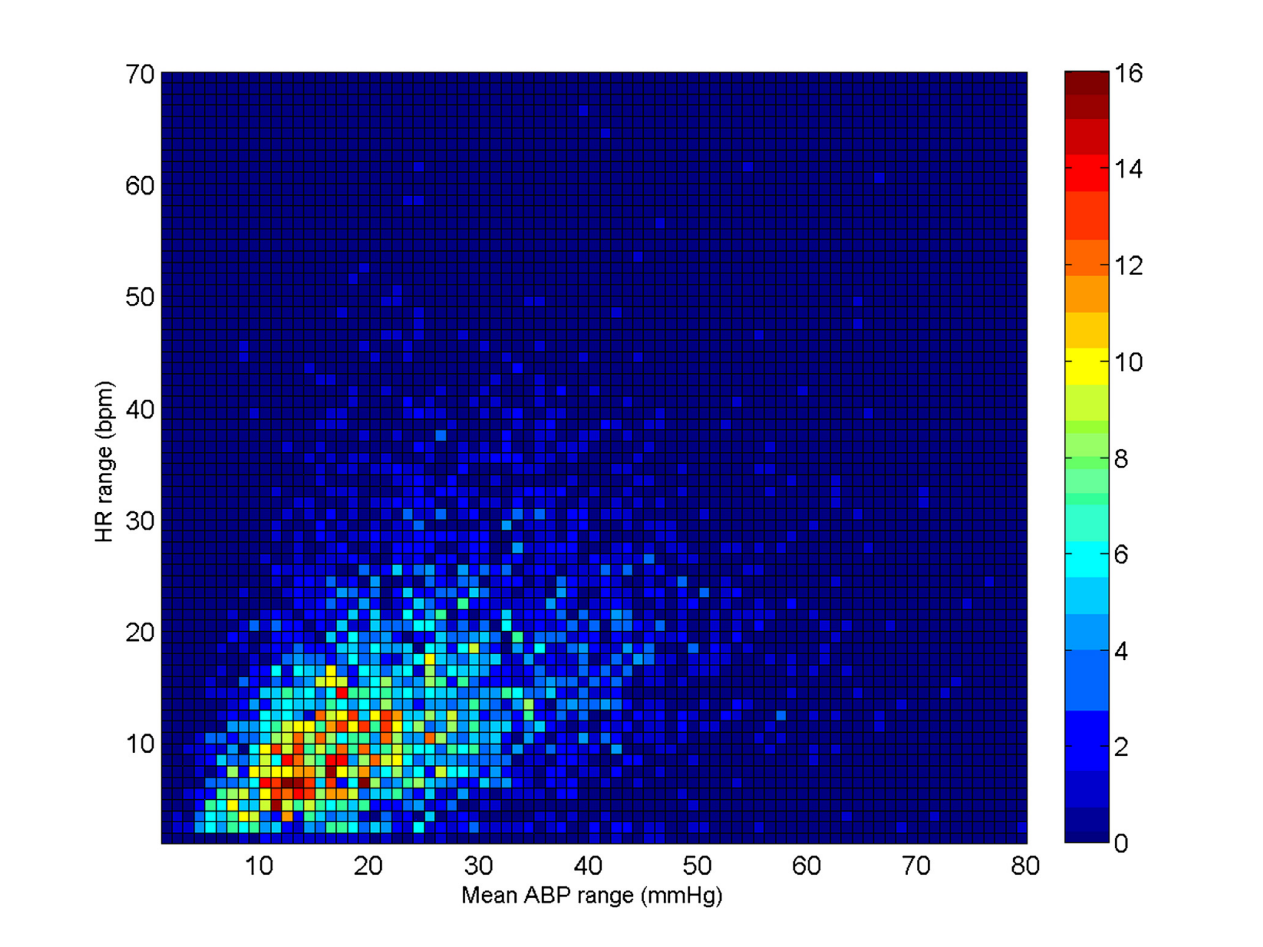
**The joint-distribution of true HR range (HR_max_-HR_min_) and BP range (MBP_max_-MBP_min_) of the clean dataset for each segment of data comprising 3762 data segments of 1 hour or longer continuous waveform data (1.62 ± 0.69 h)**.

Note also that the results for the sample-and-hold algorithm (repeating the last value when *ψ *<*ψ*_*t*_) are almost as good as the SQI-modified KF algorithm. This is in part due to the accuracy of the signal quality metric, and in part due to the fact that the data do not vary much in terms of blood pressure (or heart rate) for long periods of time. Figures 9, 10 and 11 illustrate the range of heart rates and blood pressures for the clean test data. Note that the range over all segments is approximately log-normal for HR, SBP, MBP and DBP and that most segments exhibit maximal changes of around 10 BPM and 20 mmHg. This is reflective of the fact that ICU patients are well-managed [30]. We therefore tested the algorithm on arrhythmias which exhibit rapid and large changes in both ABP and HR.

Based on previous work [6], we calculated heart rates using the median of the four shortest pulse-to-pulse (PP) intervals in the ABP waveform for tachycardic episodes, and the four longest PP intervals for bradycardic episodes [29]. Of the 707 bradycardia alarms, (506 true and 201 false), our algorithm indicated suppression of 149 of the 201 false alarms (74.1%) and only 2 of the 506 true bradycardia alarms (0.4%). For tachycardia, 233 of the 433 false alarms (53.8%) were suppressed and only 6 of the 1444 true alarms (0.4%) were suppressed.

When the ECG-derived heart rate is also used [14], and fused with the ABP-derived heart rate, we found that our algorithm indicated suppression of 167 of the 201 false extreme bradycardia alarms, and accepted 500 of the 506 true extreme bradycardia alarms. In other words, our KF-based HR estimation algorithm correctly tracked the true abnormal drops in HR 98.8% of the time, and was only 'fooled' into tracking the artifacts (that tricked the monitors into alarming) 16.9% of the time. In the case of tachycardia, only one of the 1444 true alarms was suppressed and 281 of the 433 false alarms were suppressed. That is, over 99.9% of the true episodes of extreme tachycardia were tracked correctly, and 35.1% of the false episodes were incorrectly tracked as significant heart rate increases.

To test how much the use of the ABP waveform adds to our analysis, we evaluated our KF-framework for false alarm suppression using the ECG signal alone. In such a scenario, we found that for extreme bradycardia, our algorithm suppressed only 51 false alarms and suppressed 8 true alarms by mistake and for extreme tachycardia, our algorithm suppressed 4 true alarms and only 155 false alarms.

Finally, to test the relative contributions that our signal quality metrics, and fusion approach make to the results, we repeated the arrhythmia analysis using the SH method with signal quality control. This resulted in a suppression of 146 false alarms and 2 true alarms for extreme bradycardia and the suppression of 230 false alarms 8 true alarms for extreme tachycardia. If the signal quality gating is removed, then many more true alarms are suppressed. In this scenario the results are that 155 false and 50 true bradycardia alarms are suppressed, and 399 false and 159 true tachycardia alarms are suppressed. This indicates that the signal quality analysis mainly contributes towards the improved false alarm suppression performance, although the data fusion step still provides a large increase in the false alarm suppression rate.

Clearly, fusing independent heart rate estimators from the ECG and ABP and using signal quality metrics results in a large increase in false alarm suppression rate from both single signal analysis, and conventional approaches, with only a minor (0.8%) increase in true alarm suppression rate (for extreme bradycardia) and a drop in true alarm suppression rate (from 0.4% to 0.1%) for extreme tachycardia.

## Discussion

Our artifact classification scheme, and resultant evaluations using artificial examples of each type of artifact, has highlighted the different accuracies in ABP (SBP, MBP and DBP) estimates in the presence of different types of artifacts. In general, the DBP is less noise-sensitive than the MBP, which is less noise-sensitive than the SBP. Slow saturations or decreases in pulse pressure (such as from thrombosis in the arterial line) can lead to large errors. High frequency noise appears to be less problematical in assessing accurate ABP estimates. Therefore, an automated method for classifying the type of artifacts identified in this study would be useful in tuning the response of the system, and allowing adaptation of the KF to change based upon artifact types. Such schemes could involve running assessments of the frequency content of the data [31], step-change detection and saturation detectors (such as tracking the ratios DBP/SBP and DBP/MBP).

Note that although the KF-based approach presented in this article is, on average, only marginally superior to a sample-and-hold approach when the signal quality is low, which is reflective of the infrequent changes in ABP or HR in the type of data we are using (i.e. ICU data) [30]. Of course, if a rapid change occurs during an artifactual period, the KF-fusion method is more likely (than the SH method) to accurately track the changes, as it can make use of data from other sources (such as a pulse oximeter or a non-invasive blood pressure cuff reading). This is demonstrated by the comparative results when applying our algorithm to extreme bradycardia and tachycardia with and without the addition of the ABP signal. Furthermore, the KF formulation presented in this article is a simple scalar formulation. However, it is evident that we can track a two-dimensional state ([HR, ABP]), or even a four-dimensional state ([HR, SBP, MBP, DBP]), to take advantage of the relationship between the HR and ABP. This may improve the tracking of the cardiovascular state and allow more accurate automatic rejection of erroneous estimates using our SQI-modified KF tracking procedure. However, care must be taken to factor in the subtleties of the cardiovascular changes in unhealthy patients (such as during hemorrhage), and a model of the cardiovascular system may be appropriate. Therefore, the technique presented in this paper could be extended to track cardiovascular model parameters over time, such as in Sameni *et al*. [32]. One simple extension of our approach that may be most appropriate is to the tracking of cardiac output measures, since such measures involve an estimate of the heart rate and blood pressure and require high quality waveforms. The method used in this paper may also allow automatic error bounds to be delivered with any estimate of the parameters being considered.

It is interesting to point out the generality of the results presented in this paper. The source data (the MIMIC II database) is a large ICU database consisting of a variety of patients one would expect to find in a top-level US teaching hospital. These include patient in the medical, surgical, coronary, trauma, and cardiac surgery care units. A full description of the data can be found in [18]. Data were collected using standard equipment mainly from radial arterial lines. Although it is hard to say, without analyzing large amounts of similar data, we do not imagine that changes in equipment or location would significantly affect the generality of these results. The use of arterial blood pressure lines outside of the ICU is rare (except in the operating room), and so the data we have used is likely to provide a general assessment. It is also important to consider the generality of the artifact types. While practices between countries may differ marginally, we do not imagine that this would lead to large differences in artifact types for other monitoring locations.

The work in this paper may also be extended to analyze photoplethysmograms (waveforms derived from pulse oximetery), a more common and non-invasive method of measuring the pulsatile flow in the cardiovascular system. Although excellent proprietary systems exist for signal quality assessment in such signals, and in some cases an SQI is available from the oximeter, no information is publicly available concerning the response of monitors to different artifact types, and under different recording conditions. Furthermore, no data fusion framework of blood pressure with other cardiac-related signals (such as the ECG) has been published (or, as far as we know, implemented in a practical scenario). Our approach may provide a general system for assessing the signal quality and using the SQI to automatically inform the validity of derived estimates and may be appropriate in many medical settings.

## Conclusion

We have presented a phenomenological classification system for artifact types in the blood pressure, and artificial methods for generating these artifacts. We have also presented an updated online system for continuously estimating HR and ABP using an SQI-modified Kalman Filter, and robustly weighting the estimates based on our trust in a given data segment. Using an extensive database of simultaneous ECG and ABP signals, we have evaluated our HR and ABP tracking algorithm. The proposed algorithm is shown to be robust and provides a predefined threshold (*ψ *≥ 0.9) for selecting data that may be trusted to give accurate blood pressure estimates (with an average error less than 10 mmHg). We have also demonstrated that diastolic blood pressure estimates are more robust to artifacts than mean blood pressure estimates, which in turn are more robust to artifacts than systolic blood pressure estimates. SBP estimates are likely to result in large errors for even moderate to high signal quality levels for certain types of artifact without signal quality analysis. Saturation-type noise produced the largest errors for all three blood pressure estimates. Results demonstrate that stringent signal quality measures should be used to qualify all blood pressure estimates. We have also shown that fusing independent heart rate estimators from the ECG and ABP together with SQIs in a KF framework provides a large increase in performance when tracking real episodes of extreme bradycardia and tachycardia over conventional approaches used in the modern ICU.

## Abbreviations

ABP: Arterial Blood Pressure; BPM: Beats Per Minute; DBP: Diastolic Blood Pressure; ECG: Electrocardiogram; FE: Feature Extraction; ICU: Intensive Care Unit; KF: Kalman Filter; MBP: Mean Blood Pressure; MIMIC: Multi-Parameter Intelligent Monitoring for Intensive Care; mmHg: Millimeters of Mercury (units of blood pressure); PP: Pulse-to-Pulse (interval); rMSE: Root Mean Square Error; SBP: Systolic Blood Pressure; SQI: Signal Quality Index; SH: Sample-and-Hold.

## Competing interests

The authors declare that they have no competing interests.

## Authors' contributions

GDC designed the experimental procedures, drafted the manuscript and provided overall editorial approval. GDC also provided coding assistance and a subset of the algorithms. RGM provided project guidance, interpretation of the ABP artifacts, and editorial assistance with the manuscript. QL provided descriptions of the techniques and generated the figures and editorial assistance. QL also implemented the majority of code, provided algorithm architecture input and designed some of the experiments. All authors read and approved the final manuscript.

## Supplementary Material

Additional file 1 **Matlab source code for generating artifacts described in article**. Contains the following files: bp_art.m – Matlab routine for generating artifacts sigmoid_art.m – Matlab sub-function to bp_art.m brownnoise.m – Matlab sub-function to bp_art.m sinc_art.m – Matlab sub-function to bp_art.m Click here for file
